# Application of Template Matching for Improving Classification of Urban Railroad Point Clouds

**DOI:** 10.3390/s16122112

**Published:** 2016-12-12

**Authors:** Mostafa Arastounia, Sander Oude Elberink

**Affiliations:** 1 Department of Geomatics Engineering, University of Calgary, 2500 University Drive NW, Calgary, AB T2N 1N4, Canada; 2 Faculty of Geo-Information Science and Earth Observation (ITC), University of Twente, P.O. Box 217, Enschede 7514 AE, The Netherlands; s.j.oudeelberink@utwente.nl

**Keywords:** LiDAR, point cloud, object recognition, segmentation, rail, cable

## Abstract

This study develops an integrated data-driven and model-driven approach (template matching) that clusters the urban railroad point clouds into three classes of rail track, contact cable, and catenary cable. The employed dataset covers 630 m of the Dutch urban railroad corridors in which there are four rail tracks, two contact cables, and two catenary cables. The dataset includes only geometrical information (three dimensional (3D) coordinates of the points) with no intensity data and no RGB data. The obtained results indicate that all objects of interest are successfully classified at the object level with no false positives and no false negatives. The results also show that an average 97.3% precision and an average 97.7% accuracy at the point cloud level are achieved. The high precision and high accuracy of the rail track classification (both greater than 96%) at the point cloud level stems from the great impact of the employed template matching method on excluding the false positives. The cables also achieve quite high average precision (96.8%) and accuracy (98.4%) due to their high sampling and isolated position in the railroad corridor.

## 1. Introduction

Rail transportation is one of most popular means of traveling all over the world. For example, 22.7 billion passengers in Japan use the rail transportation every year. Rail freight is also a very common means of goods transportation. The rail freight of Russia and the USA, for instance, constitute up to 65% and 42% of their total freight, respectively [1]. Furthermore, 15% of all accidents in the USA in 2014 were rail-related [2]. This indicates that the safety of the railroad environments is a crucial issue. Three primary causes of the rail-related incidents are human mistakes, rail defects, and equipment failure. Due to the safety restrictions, a certain clearance is required between the railroad assets and the surrounding environment, which is sometimes violated by the surrounding eco-system. A tree root destabilizing the track bed and unchecked grown vegetation are two instances that can breach such clearance and endanger the safety of trains in motion.

To address such safety concerns, the railroad environments are regularly monitored by staff that traverse along the railroad corridor and visually inspect it. For instance, the federal railroad administration (FRA) of the USA mandates the rail tracks to be examined twice per week [3]. However, the manual inspection is incompetent due to the associated high expenses, low pace, and human fallibility. The efficiency of railroad monitoring can be significantly enhanced by employing mobile laser scanning (MLS) systems that provide a precise and accurate three-dimensional (3D) representation of the current state of the railroad environment. A MLS system provides quite fast data acquisition and very precise 3D measurements. However, MLS data are typically quite large and the key to exploit such large-volume data is the automation of data processing.

This study aims to develop automated methods to recognize railroad assets from MLS data in support of the railroad corridor monitoring. The result of this work can be employed to identify a problematic eco-system and the potential defect in the railroad assets. The problematic eco-systems may appear as a giant tree root, de-stabilizing the rail tracks. The proposed methodology is a knowledge-based approach that takes advantage of very basic knowledge of the urban railroad configuration that is the spatial offset among railroad assets. The algorithm is an integrated data-driven and model-driven method so that the advantages of both types of methods are employed. The efficiency of data-driven methods is higher than the efficiency of model-driven methods. However, the performance of model-driven methods for poorly sampled datasets is better than that of data-driven methods.

## 2. Literature Review

A MLS system consists of two primary units: an imaging unit; and a navigation unit. The imaging unit is composed of one or multiple Light Detection And Ranging (LiDAR) sensor (s) that collect data by a laser range finder system. A LiDAR sensor emits a laser beam and measures the range by computing the time of flight or the phase change of the returned beam. Afterwards, the 3D coordinates of each and every point are calculated by the measured range and the orientation of the laser deflecting mirror. In mobile mapping, the imaging unit is attached to a moving vehicle and collects 3D geometrical information in two-dimensional (2D) profiles along the vehicle’s trajectory [4] and the collected data would be in a local coordinate system centered on the location of the LiDAR sensor at the time of scanning. A global navigation satellite system (GNSS) and an inertial navigation system (INS) are two components of a navigation unit that is employed for geo-referencing of the data acquired by the imaging unit. The optical remote sensing data collected by the imaging unit and the spatial data acquired by the navigation unit are then integrated and the resulting data is a registered point cloud that is points with 3D coordinates in a national or global coordinate system. Puente et al. [5] and Petrie [6] introduce different MLS systems with different sensors and configurations.

Many works in recent years have studied the extraction of objects from point clouds of various environments such as urban, road, and underground environments [7,8,9,10]. The following reviews the most relevant studies in object extraction from railroad corridor point clouds. Morgan [11] visually investigates the current state of the railroad corridors from MLS data. Leslar et al. [12] and Soni et al. [13] employ manual methods to model rail tracks from MLS data and terrestrial laser scanning (TLS) data, respectively. References [11,12,13], however, do not propose any systematic or automated approach. Beger et al. [14] reconstruct the rail tracks centerline by integrating airborne laser scanning (ALS) data and extremely high resolution ortho-imagery. They derive the rail tracks mask from ortho-imagery by utilizing image processing edge detection algorithms. Then, points belonging to the rail tracks are classified using the spatial information provided by the rail track masks. Beger et al. [14] propose an improved and more automated version of the algorithm developed by Neubert et al. [15] who use a pre-classified data. Sawadisavi et al. [16] use imagery and machine vision technology to identify the irregularities and defects in wood-tie fasteners, rail anchors, crib ballast, and turnout components. Zhu and Hyyppa [17] recognize the terrain, roads, buildings, and trees in the surroundings of the railroad environment. They integrate ALS data and MLS data and then convert the integrated data to images and employ image processing techniques. The primary railroad components such as rail tracks are not identified by Sawadisavi et al. [16] and Zhu and Hyyppa [17] though.

The following discusses in great detail the shortcomings of the authors’ previous contributions in this field as well as the novelty and improvements in this study. Elberink and Khoshelham [18] employ a data-driven and a model-driven approach to approach to extract rail tracks and 3D models to determine the centerline locations. Oude Elberink and Khoshelham [18] extend their earlier work in Oude Elberink et al. [19], which only handles the detection and modelling of rail tracks. However, the approach proposed by Oude Elberink and Khoshelham [18] is computationally very intense since:

1. For the detection of rail tracks and their centerlines a point-wise calculation is employed to find potential rail track points and its corresponding parallel rail track.
2. It applies the Least Squares adjustment to each and every piece of rail track for the model fitting part.
3. The utilized Least Squares adjustment is a non-linear model, which needs to be linearized and run in many iterations in order to deliver acceptable results.
4. The Fourier series is applied (after modeling) to enforce the smoothness in the rail tracks’ shape, which imposes even more computational load.

The above four points clearly indicate how computationally expensive the methods proposed by Oude Elberink and Khoshelham [18] are, although the last three deal with the 3D model fitting part which is not part of the current research. Arastounia [20] extracts key components of rural railroad corridors using a data-driven approach. Although railroad infrastructure is successfully recognized, the employed dataset contains a simple configuration of a rural railroad environment. Herein, a dataset of an urban railroad corridor is employed that includes more diverse features such as humans, cars, and buildings in a much denser configuration. The data-driven approach suggested by Arastounia [20] generates good results on a very well sampled dataset whose point sampling is almost twice as dense as sampling of the data used in this contribution. The proposed methods by Arastounia [20] degenerate poor results on the dataset used in this contribution due to its low sampling rate and complicated configuration. Furthermore, Arastounia [20] identifies points belonging to rail tracks by seeking local 3D spherical neighborhoods in the entire dataset, which is quite time-consuming. In this contribution, points belonging to rail tracks are identified by fitting a 2D grid to the track bed and investigating the height variation within each grid cell, which significantly decreases the computational load. That being said, herein, a data-driven algorithm is developed that employs points’ local neighborhood distribution in 3D space (including the height information) for the initial classification of rail tracks. This is followed by a customized template matching algorithm for the elimination of rail track false positives by considering the topological relationship among rail tracks and cables. The template matching uses a simple equation (Equation (1)), without the need for a point-wise attribute calculation as described in Oude Elberink and Khoshelham [18]. Therefore, the proposed methodology in this contribution takes advantage of both data-driven and model-driven methods that enable it to handle poorly sampled datasets with complex configurations while preserving a high computational efficiency. The proposed algorithm also only requires point clouds with no other sources of information such as intensity or RGB data.

## 3. Methodology

**Figure 1:**
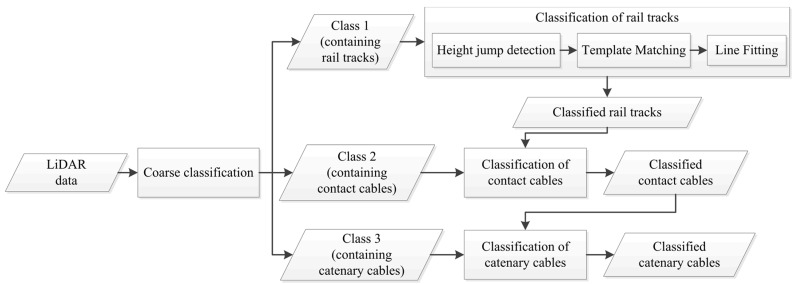
Flowchart of the proposed methodology.

Section 3.1 introduces the objects of interest and developed algorithm is then described in the following three sub-sections. First, points are coarsely classified based on their height in Section 3.2. Next, points belonging to rail tracks are identified in Section 3.3 and points belonging to overhead cables are classified in Section 3.4. Figure 1 indicates the flowchart of the developed algorithm.

### 3.1. Objects of Interest

**Figure 2:**
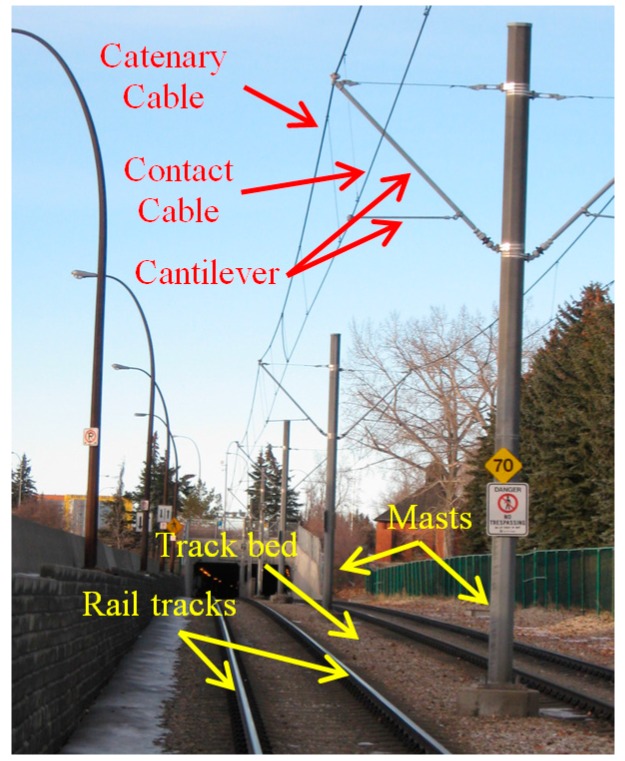
Components of an urban railroad corridor.

Figure 2 shows a sample urban railroad environment in which the railroad assets are indicated by arrows. The railroad assets are described in the following.

- Track bed: is the surface underlying rail tracks and consists of a sub-grade level topped with ballast, which holds rail tracks in line and on the surface. Ballast consists of sized hard particles easily handled in tamping, which distribute the load, and drain well [3]. Although the track bed is not a key element of the railroad infrastructure, it defines the areal extent of the railroad corridor.
- Rail tracks: are composed of two parallel rolled-shaped steels that provide a stable platform for trains in motion. The dimensions of rail tracks are the same in the entire railroad corridors of each country and the neighboring countries so that the railroad corridors of the neighboring countries can be interconnected.
- Masts: are posts that are located on the track bed to hold the overhead cables in place. They are usually made from wood or metal.
- Contact cables: appear as straight linear objects that lie at the lowest height among all overhead cables and transmit the power to the trains.
- Catenary cables: appear as curvilinear objects that lie immediately above the contact cables and hold the contact cables in place. Catenary cables are interconnected with masts by thin metal tubes called cantilevers.

In this work, the objects of interest are rail tracks, contact cables, and catenary cables, which constitute the salient elements of the railroad environments. The detailed description of railroad infrastructure can be found in Bianculli [3].

### 3.2. Coarse Classification

**Figure 3:**
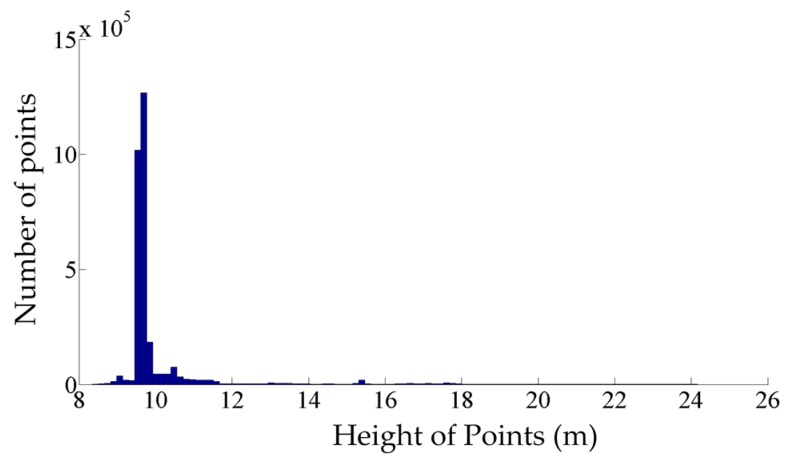
Histogram of points’ height of the entire dataset.

The coarse classification is carried out based on the points’ height so that each class contains only one type of the objects of interest. To that end, the height of the track bed is found by seeking the most common height in the dataset. Since the track bed has the largest dimensions among all components of the railroad environment, the most common height represents the height of the track bed. Figure 3 shows a sample height histogram of a railroad point cloud in which the largest peak represents the track bed height. Then, points within half a meter higher or lower than the track bed height are clustered in class 1. As a result, the class 1 comprises all points on the track bed and rail tracks. Afterwards, points whose height is within 5 m and 5.5 m above the track bed are classified in the second class, which includes points belonging to the contact cables. Next, points that lie higher than 5.5 m above the track bed are put into class 3, incorporating points belonging to the catenary cables.

As the title of this article suggests, the proposed algorithm is primarily targeting the urban railroad corridors in which, due to safety regulations, the track bed typically does not have a large slope even over long distances. This implies that the height difference among the track bed and over-head cables remains constant and the coarse classification based on points’ height is applicable. The railroad corridor in rural areas might experience a large slope in rare circumstances such as in mountainous areas. For such environments, the coarse classification can be applied to portions of the datasets where the height difference among railroad key elements remains within the employed thresholds. It is required to note that the remaining parts of the developed algorithm are applicable to both urban and rural railroad corridors regardless of the track bed’s slope.

The coarse classification significantly improves the efficiency of the data processing since, as a result of this step, points belonging to the rail tracks, contact cables, and catenary cables are separated from one another and are clustered in separate classes. Therefore, only points of class 1 need to be processed to identify the points belonging to the rail tracks, only points of class 2 should be analyzed to classify the points belonging to the contact cables, and only points of class 3 are required to be inspected to cluster the points belonging to the catenary cables.

### 3.3. Classification of the Rail Tracks

The points belonging to the rail tracks are classified in three steps: the identification of candidate rail points; excluding incorrect rail track recognitions (false positives) and including the not-classified rail points (false negatives).

**Figure 4:**
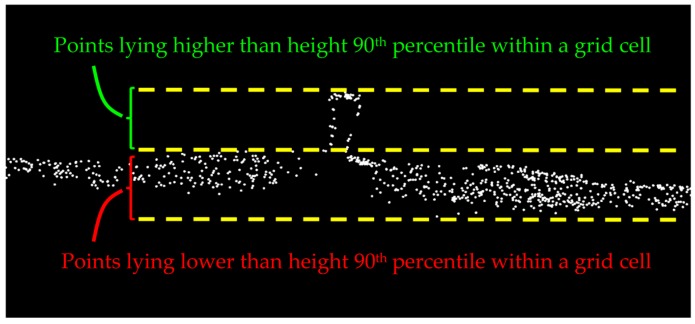
The height 90th percentile threshold is utilized for the initial classification of points belonging to rail tracks within each grid cell.

The candidate rail points are identified by detecting the height jumps on the track bed. To that end, a 2D planimetric grid is fitted to the points of class 1 and the grid cells with height variation as large as the rail track height (0.1 m) are marked. The height variation of each grid cell is achieved by computing the height difference between its height 5th and 95th percentiles so that the noise and outliers are excluded. Then, points higher than the height 90th percentile of the remaining outlier-free points within each marked grid cell are labeled as the candidate rail points. The 5th and 95th percentile thresholds are selected with respect to the dataset’s noise level. The primary source of the noise in railroad corridor point clouds is multipath whose associated noise cannot be quantified. Multipath occurs once a laser beam returns to the LiDAR sensor from more than one straight path, due to being reflected by the highly-reflective neighboring surfaces such as adjacent steel rail tracks and metal masts. Quantifying the multipath-introduced noise requires tracing the path of each and every laser beam from the moment it is emitted by the LiDAR sensor until it returns to the sensor, which is obviously infeasible. Therefore, the dataset’s noise level is empirically inspected for the selection of height percentile thresholds in the outlier elimination step. The 90th percentile threshold used for distinguishing rail tracks from the underlying track bed is chosen by considering the much larger dimensions of the track bed compared to rail tracks. This implies that points belonging to the underlying track bed constitute a large portion (90%) of points within each grid cell that lie in a lower height and the remaining (10%) points belong to the atop rail track (Figure 4). The grid cell size is 0.5 m × 0.5 m, considering the dataset point sampling and the spatial offset between rail tracks (gauge).

**Figure 5:**
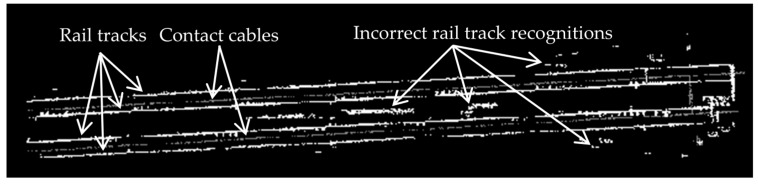
The greyscale image of the candidate rail tracks (represented in white color) and points of class 2 (depicted in grey color).

Some incorrect rail track identifications are expected due to the height jumps induced by non-rail track objects on the track bed. Such incorrect recognitions are excluded by employing a model-driven approach (template matching), which is an image processing technique. The template matching starts by converting the candidate rail points and all points of class 2 into a greyscale image whose resolution is 0.1 m; that is each pixel represents a 0.1 m × 0.1 m area of the actual railroad environment. The pixels’ value can be 0, 125, or 255 representing black, grey and white color, respectively. The greyscale image is generated by projecting the candidate rail points and all points of class 2 onto a planimetric plane (ignoring their Z components). Then, the location of each point in the greyscale image is determined. The cells with candidate rail points are shown in white color, cells with points of the second class are depicted in grey color, and the remaining cells are shown in black color. Figure 5 demonstrates a sample greyscale image, in which two pairs of rail tracks appear as four straight white lines and two contact cables appear as two grey lines between each pair of rail tracks. The other white areas in this figure represent the incorrect rail track recognitions, which need to be excluded.

**Figure 6:**
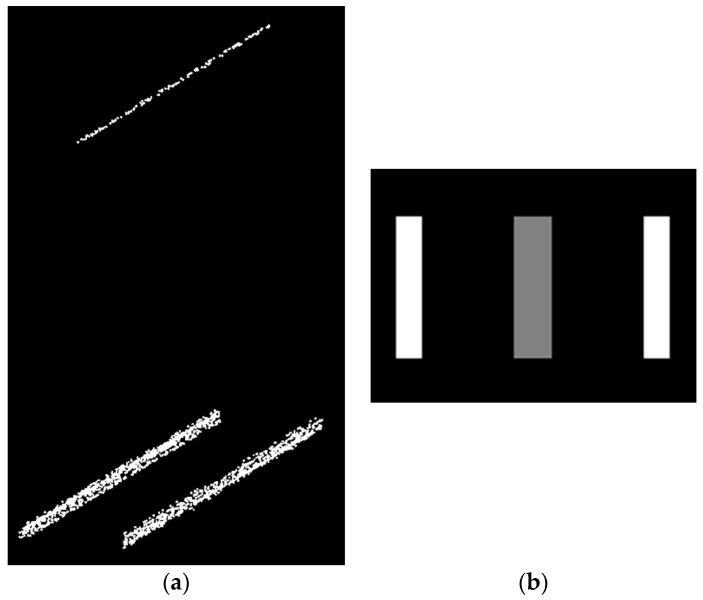
The template: (**a**) The template point cloud; (**b**) Its corresponding greyscale image including one meter of a pair of rail tracks and one meter of a contact cable atop in which the rail tracks are depicted in white color and the contact cable is shown in grey color

Afterwards, a template of one meter of the railroad corridor is created by converting its point cloud into a greyscale image. The template point cloud includes one meter of a pair of rail tracks with one meter of a contact cable atop, which is obtained from the original point cloud. The spatial offset between two rail tracks in the template point cloud is fixed and in plan view, and the cable is located approximately on the center of the rail track pair. The conversion is carried out in the same way as the greyscale image was created. Figure 6a indicates the template point cloud and Figure 6b depicts its corresponding greyscale image. Similar to Figure 5, in Figure 6b, the white and grey colors represent the rail tracks and contact cables, respectively. It is required to note that, in plan view, the location of the contact cable may be slightly deviated from the rail tracks’ centerline. That being said, the grey part in Figure 6b (representing the location of the contact cable) is considered to be marginally wider than the cables’ width in order to recognize the contact cable located slightly apart from and on either side of the rail track’s centerline. This consequently makes the developed algorithm applicable to a broader range of datasets since the cables are not required to be located exactly on the centerline of a rail track pair, in plan view.

The correlation between the template (Figure 6b) and every pixel of the greyscale image (Figure 5) is calculated. In the developed methodology, no assumption is made and no a priori knowledge is used regarding the orientation and direction of the rail tracks. So, the template is rotated from −90° to +89° with 1° steps and the correlation between each pixel and every rotated version of the template is calculated to ensure rail tracks with any orientation are recognized. The correlation is calculated using the following equation, which denotes a normalized correlation measure. In this equation, x represents the location of template’s center on greyscale image and N is 5 denoting half size of the template, which is computed with respect to the template size in real world (1 meter) and image resolution (0.1 m). T(i) and I(x+i) also indicate the value of template and greyscale image on the query pixel, respectively. The correlation measures consequently range from zero (corresponding to no correlation) to one (corresponding to a total correlation).

(1) $$\text{Correlation}(x)=\frac{\sum_{i=-N}^{N}\left(T(i)\times I(x+i)\right)}{\sqrt{\sum_{i=-N}^{N}{\left(T(i)\right)}^{2}}\times \sqrt{\sum_{i=-N}^{N}{\left(I(x+i)\right)}^{2}}}$$

**Figure 7:**
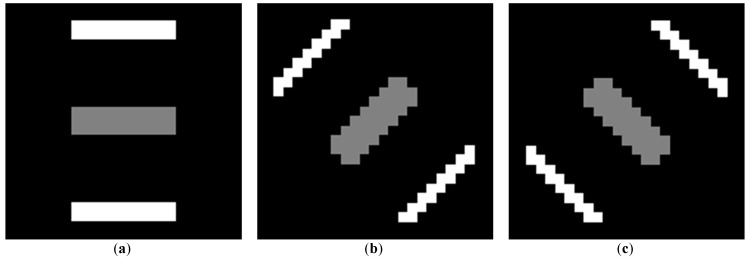
Three sample rotated templates: (**a**) Rotated by −90°; (**b**) Rotated by −45°; (**c**) Rotated by +45°.

**Figure 8:**
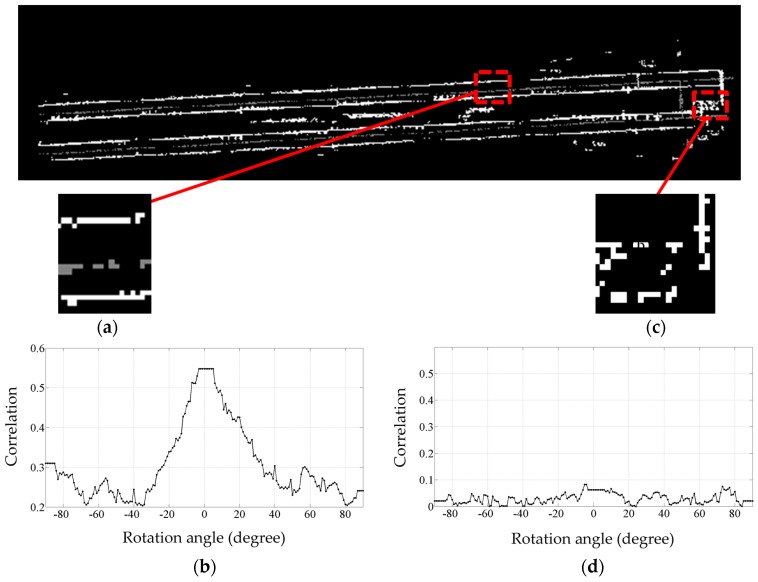
The results of template matching in two different parts of the dataset with the template rotated from −90° to +89°: (**a**) A sample part that contains a piece of rail track; (**b**) Its correlation plot indicating a distinctive peak at 0°; (**c**) A sample incorrect rail track recognition; (**d**) Its correlation plot showing no peaks for any rotated version of the template.

Once the correlation exceeds a certain threshold (in this case 0.5), the pixel under inspection is considered to contain true rail track points. If the correlation between the template and a pixel with candidate rail points does not exceed the threshold, the candidate rail points are not considered for further processing. Figure 7 indicates three sample rotated versions of the template. Figure 8 indicates two instances of template matching results. Figure 8a depicts a sample part with a piece of rail track and Figure 8c shows a sample incorrect rail track recognition that is identified and excluded by template matching method.

The correlation threshold is chosen in regards to the dataset’s point sampling, noise level, and grid cell size, corresponding to the resolution of the grey scale image of the data and template. As is evident in Figure 8b,d, the selected threshold (0.5) is not a critical value since the correlation of grid cells without a piece of rail track is much lower than that of cells containing a piece of rail track.

To enhance the computational efficiency of template matching, once the first one meter of rail tracks is recognized, the direction of each rail track pair is calculated and considered as the “estimated direction” of the following one meter of the query rail track. Thus, to identify the next one meter of the rail track, the template is rotated from the estimated direction −20° to estimated direction +20° since the rail tracks’ smooth curvature gradient requires their direction to not dramatically change within such a small length (one meter), due to safety regulations. The “estimated direction” of the rail track pair is updated as the following one-meter piece is detected.

To include not-classified rail points, a line is fitted to one meter pieces of rail tracks and the pixels lying on the fitted lines are classified as rail tracks. The line fitting is based on the fact that the rail tracks can be considered straight on short length since their curvature on short length is quite minimal. The result of this section is a greyscale image whose white pixels represent points belonging to rail tracks. Afterwards, the greyscale image is converted back to 3D point cloud. To this end, points that are located on the final white pixels are considered as belonging to the rail tracks.

### 3.4. Classification of the Overhead Cables

The overhead cables consist of two types of cable: the contact cable; and the catenary cable. The contact cables are classified by employing their topological relationship with the rail tracks that is, in top view, contact cables appear as a straight linear object between each pair of rail tracks. Additionally, due to the safety restrictions in the railroad corridors, there are no other objects between the rail tracks and the contact cables. That being said, the rail tracks identified in the previous step and all points in the second class are projected onto a planimetric plane and only points of the second class that are within one meter neighborhood of the projected rail points are considered as belonging to the contact cables. The one meter threshold is selected by considering the location of each contact cable between a pair of rail tracks in top view and the spatial offset between two rail tracks (gauge), which is about 1.4 m.

Catenary cables are attached to contact cables at regular intervals by vertical wires called droppers, implying that catenary cables are located precisely above contact cables. Therefore, points classified as the contact cables in the previous step and all points of class 3 are projected onto a planimetric plane and only points of the class 3 that are within 0.2-m Euclidean distance of the projected contact cables are classified as catenary cables. The planimetric distance threshold (0.2-m) is selected based on the cables thickness and the topological relationship between the contact cables and the catenary cables.

**Figure 9:**
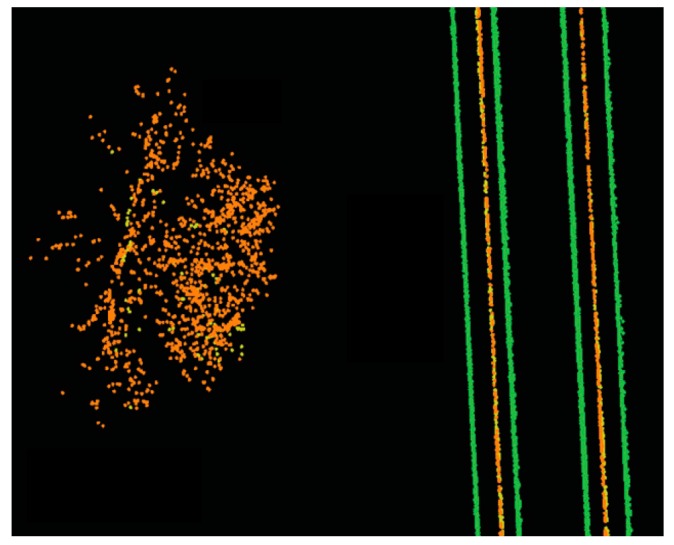
Topological relationship among railroad infrastructure in which points belonging to rail tracks, class 2, and class 3 are depicted in green, yellow, and brown color, respectively. When all points are projected onto a planimetric plane, the overhead cables appear as straight linear objects between each pair of rail tracks and the catenary cables lie exactly on top of contact cables.

It is required to note that incorrect classifications in the results of catenary cables (at intersects of the masts and catenary cables) are expected. The topological relationship among the railroad infrastructure is indicated in Figure 9 in which points belonging to the rail tracks, class 2, and class 3 are depicted in green, yellow, and brown color, respectively. As it can be seen in this figure, when all points are projected onto a planimetric plane, the overhead cables appear as straight linear objects between each pair of rail tracks. Moreover, Figure 9 shows that catenary cables lie exactly on top of the contact cables in top view. This indicates that the catenary cables are the only objects of class 3 that is in a very close planimetric neighborhood of contact cables. The distance of other objects of class 3 (like the tree shown in brown color) is much more than the distance of the catenary cables from the contact cables.

## 4. Dataset

**Figure 10:**
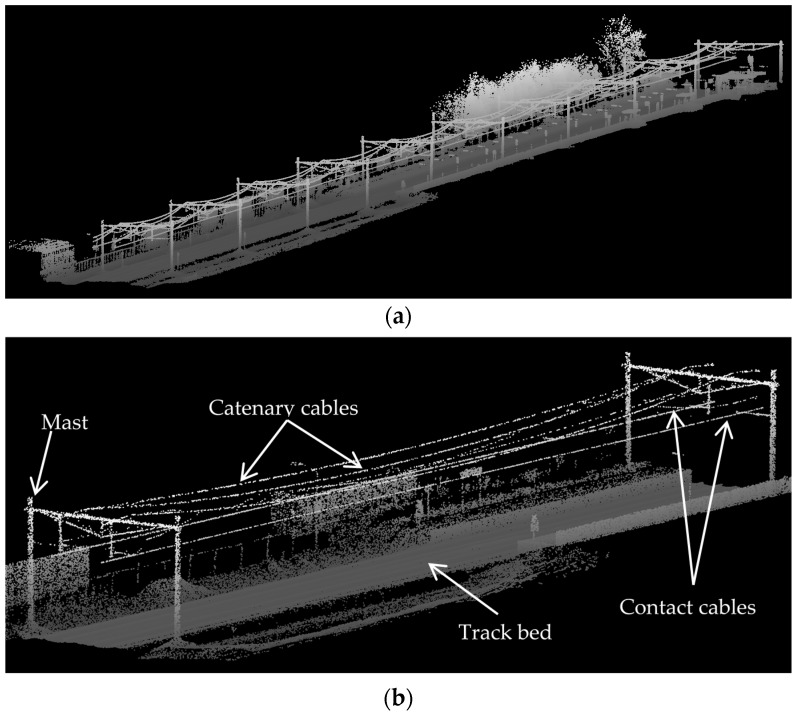
The dataset is depicted from two different views: (**a**) The entire dataset from an oblique view; (**b**) A finer representation of the railroad assets in this dataset.

**Figure 11:**
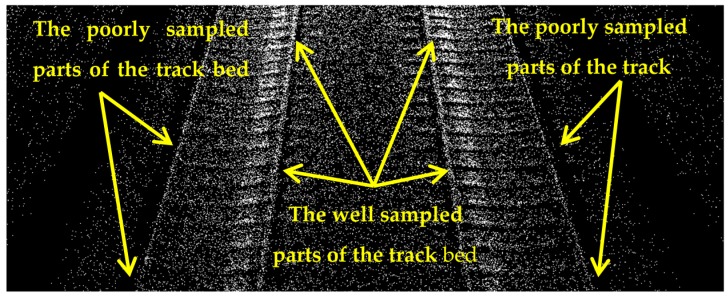
The non-uniform point sampling on the track bed. The point sampling around the inner rail tracks are much higher than that around the outer rail tracks.

The dataset used in this contribution is collected by mounting an MLS system on an open-top rail car and scanning the railroad environment when the train was in motion. The employed MLS system was set to scan the railroad corridor with a sampling that makes the objects of interest identifiable. The captured dataset covers about 630 m of railroad corridor near the city of Elst in the Netherlands. It has more than 3 million (3,079,210) points, containing only 3D coordinates of points in the national coordinate system of the Netherlands and the precision of the points’ coordinates are in millimeter level. The dataset comprises two pairs of rail tracks, two contact cables, two catenary cables, ten masts, two train stations, the surrounding vegetation, building facades, humans, etc. Figure 10a portrays an oblique view of the entire dataset and Figure 10b indicates a finer representation of the railroad assets in this dataset. As is evident in these figures, the 10 masts are located in regular spatial intervals (approximately 67 m) and catenary cables appear as two pairs, each pair lies immediately above a contact cable and the cables of each pair intersect where they reach a mast. The point sampling is not homogeneous in different parts of the dataset, especially on the track bed. As is evident in Figure 11, the track bed sampling around the inner rail tracks is almost 2.5 times as dense as the track bed sampling around the outer rail tracks. This is due to the scanning pattern of the employed MLS system and the orientation of its LiDAR sensors. Table 1 presents the average point sampling on the objects of interest including two numbers for the sampling of the track bed: one number for the well sampled area around the inner rail tracks; and one number for the poorly sampled areas around the outer rail tracks. The point sampling of the contact cable and catenary cables are much lower than that of the track bed due to smaller dimensions of the cables, compared to the track bed. The sampling of contact cables is also higher than the sampling of catenary cables since contact cables lie in a closer spatial offset from the LiDAR sensors.

## 5. Results and Discussion

**Figure 12:**
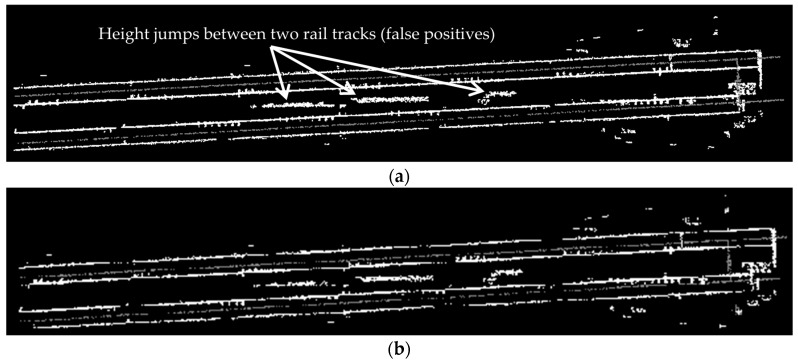

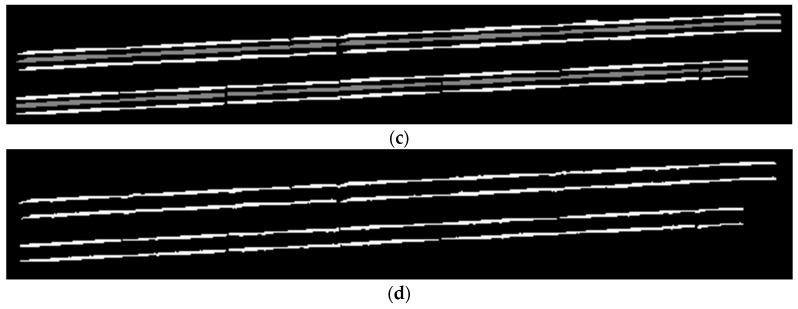
The intermediate and final results of the employed template matching method: (**a**) The point cloud of the height jump detection results in which height jumps and points of class 2 are indicated in white and grey color, respectively; (**b**) The grey scale image of the height jump results; (**c**) The template matching results in which the false positives in the rail track classification results are excluded; (**d**) The template matching final result that shows only the rail tracks.

Figure 12 presents the intermediate steps and results of the template matching method. Figure 12a indicates the point cloud of height jump detection results and Figure 12b depicts its corresponding greyscale image. Figure 12c shows the template matching result in which the false positives are excluded and Figure 12d demonstrates the final result that includes only the rail tracks. The grey color in all parts of this figure represents the points of class 2 and the white color in Figure 12a,b indicates the height jumps that may be induced by the rail tracks or external objects. The white color Figure 12c,d represents the height jumps that are caused only by the rail tracks.

**Figure 13:**
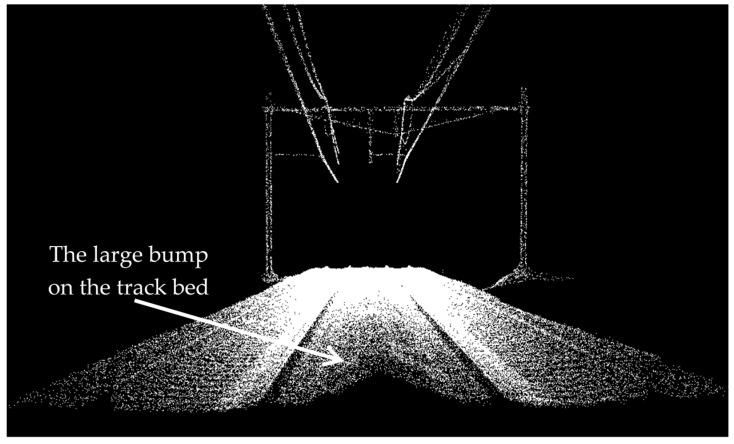
The presence of a large bump on the track bed induces some false positives in the results of height jump detection.

**Figure 14:**
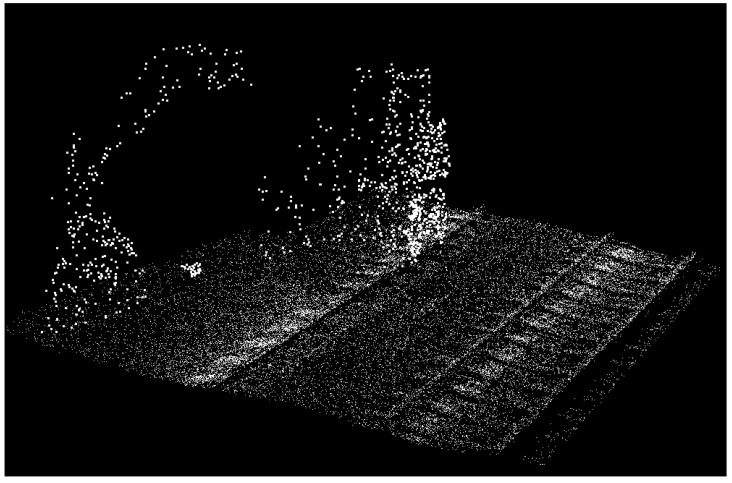
An external object on the rail tracks prevented the complete data collection on some parts of the rail tracks.

As is evident in Figure 12a, many height jumps are identified between two pairs of rail tracks. This is due to the track bed configuration in this dataset in which there is a large bump between two pairs of rail tracks (depicted in Figure 13). Moreover, as it can be visualized in Figure 12d, the last few meters of the lower pair of rail tracks are not identified. This is due to the presence of a large external object on the rail tracks during data collection, which is indicated in Figure 14. The last few meters of not-classified rail tracks are recognized by fitting lines to the classified rail tracks, as described in the last paragraph of Section 3.3.

**Figure 15:**
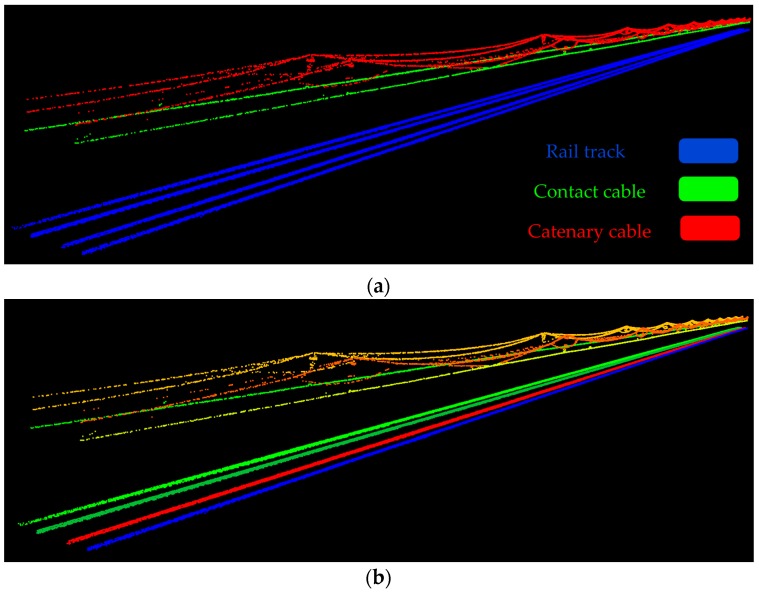
Results: (**a**) The classification results based on the object type; (**b**) The classification results in which all objects are separately recognized and demonstrated in a different color, regardless of their type.

Figure 15 indicates the classified railroad components. Figure 15a indicates each type of object in a different color and Figure 15b depicts each object separately in a different color, regardless of its type.

The results are analyzed in terms of the precision and accuracy both at the object level and at the point cloud level. To that end, points on the objects of interest were manually extracted and were utilized as ground truth data. The precision and accuracy are computed as in Equations (2) and (3).

(2) $$\text{Precision}=\frac{tp}{tp+fp}$$

(3) $$\text{Accuracy}=\frac{tp+tn}{tp+tn+fp+fn}$$

where tp, fp, tn, and fn represent true positive, false positive, true negative, and false negative, respectively. Table 2 presents the classification results at the object level and Table 3 presents the results at the point cloud level. As is evident in Table 2, all railroad components including four rail tracks, two contact cables, and two catenary cables are successfully classified at the object level with zero false negatives and zero false positives. This corresponds to 100% precision and 100% accuracy at the object level.

**Figure 16:**
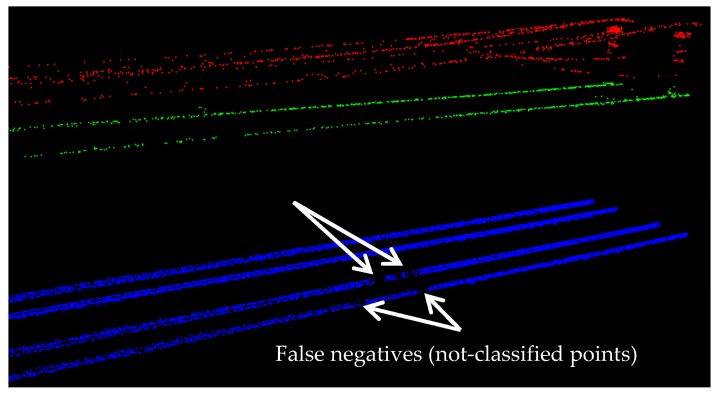
False negatives (not-classified points) in the rail tracks classification results are indicated by arrows. This is due to the presence of an external object on the rail track.

The average precision and average accuracy of all objects at the point cloud level are quite high (greater than 97%). The classification of rail tracks is the most challenging part of this work since they have small vertical dimensions (about 10 cm high) and they are surrounded by many other objects in their close neighborhood such as the wood-tie fasteners, rail anchors, and crib ballast. Despite that, the classification of rail tracks is quite successful since it achieved both high precision (98.4%) and high accuracy (96.5%). This clearly highlights the key role of the employed model-driven approach (template matching) in excluding the vast majority of false positives in the classification results of the rail tracks. The remaining false positives are quite minimal and are all attached to the rail tracks. In fact, the rail tracks false positives are limited to a very small portion of the track bed in a very close neighborhood of the rail tracks. Moreover, as it was mentioned in Section 4 (dataset), the point sampling in different parts of the track bed is quite fluctuant. This stresses the capability of the applied model-driven method in delivering good results even for poorly sampled parts of the dataset while the performance of data-driven methods is highly dependent on the dataset point sampling. Additionally, considering that the classification of cables is carried out based on the classified rail tracks, the exploited model-driven method has an indirect positive impact on the cables classification results as well. Furthermore, the lower accuracy of rail tracks (compared to that of the cables) is due to the presence of an external object on the rail tracks during the data acquisition, shown in Figure 14. As a result of this external object, small gaps (areas with no point) exist on parts of the rail tracks where the external object was located. Theses gaps (shown in Figure 16) resulted in non-perfect classification accuracy of rail tracks.

The contact cables have the highest precision and accuracy at the point cloud among all objects since: they are relatively well sampled (considering their very thin structure); and they have an isolated position in the railroad corridor. This is evident in Table 1 that indicates the average sampling of contact cables is almost twice as dense as the average sampling of the catenary cables. Considering that a data-driven method is employed to classify cables, the higher sampling of contact cables (compared to that of the catenary cables) explains the more successful classification of contact cables. Furthermore, the isolated position of the contact cables plays a crucial role in their very high precision and accuracy.

**Figure 17:**
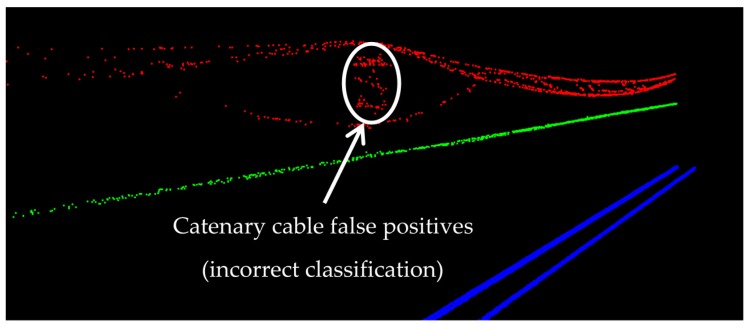
False positives in the classification results of catenary cables at their intersection with masts.

The catenary cables have the lowest precision since they are interconnected with the masts that hold the cables in place. As a result, small parts of the masts at the intersections with catenary cables are incorrectly classified as catenary cables (false positives), which is demonstrated in Figure 17. The high accuracy of catenary cables (almost 98%) is primarily because they are the only objects located immediately above the contact cables. So, once the contact cables are classified, the catenary cables can be classified by seeking for the points above contact cables and in a close planimetric neighborhood of contact cables.

**Figure 18:**
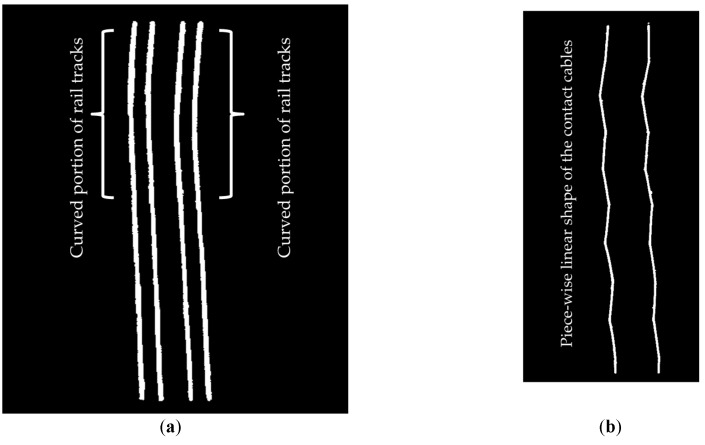

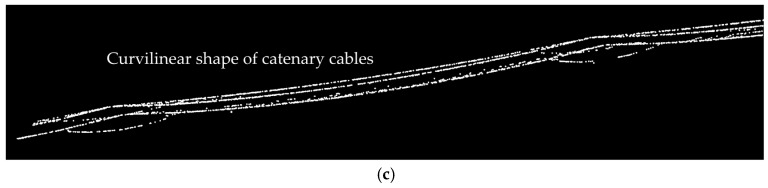
The physical shape of the objects of interest: (**a**) The curvature of rail tracks is indicated from plan view; (**b**) Piece-wise linear shape of the contact cables is shown from plan view; (**c**) The curvilinear shape of catenary cables is depicted from an oblique view.

The dataset contains many diverse urban features such as building facades, lamp posts, humans, and trees, which makes the classification of railroad assets more challenging. However, the configuration of railroad assets is not very complicated since it is composed of two pairs of rail tracks and four overhead cables with no merging rail tracks and no intersecting cables. However, rail tracks experience a smooth curvature in their second half (Figure 18a). This indicates that the developed algorithm is able to classify both straight and curved rail tracks. The contact cables have a piece-wise linear shape (Figure 18b) that is composed of interconnected linear segments. The catenary cables have a curvilinear shape (Figure 18c) and each catenary cable is split into two cables and the cables of each pair intersect at their intersection with the masts. The cable classification results imply that the proposed methodology is capable of recognition of the cables in spite of their complicated shape since the topological relationship between the cables and rail tracks (and not the shape of the cables) are utilized for their recognition.

The following inspects the efficiency, sensitivity, automation, and robustness of the developed algorithm. As it was mentioned in Section 3, the data driven methods are computationally more efficient than model-driven approaches. However, the performance of model-driven approaches is better on poorly sampled parts of the data, compared to the performance of data-driven methods. That is why a hybrid method composed of a data-driven method and a model-driven method is developed in this work so that the algorithm efficiency is boosted by the data-driven method and the poorly sampled parts of the data are handled by the model-driven method. However, the performance of the algorithm is highly sensitive to its performance in the previous steps. That is the contact cables are classified by employing the rail tracks classified in the first step of the methodology and the catenary cables are classified by utilizing the contact cables identified in the second step of the methodology. Thus, false positives (incorrect classifications) of rail tracks directly results in the false positives in the classification of contact cables and false positives of contact cables induces false positives in the catenary cables. However, herein, as a result of employing template matching method, the rail track false positives are so minimal that they do not introduce any false positives in the classified contact cables. Similarly, no false positives are introduced in the classified catenary cables since the false positives of the contact cables are also quite minimal.

Furthermore, the developed algorithm is fully automated and quite robust since it makes no assumption and does not use any a priori knowledge regarding the location, orientation, and curvature of the objects of interest. The employed template matching method is able to handle any orientation and any degree of curvature of the rail tracks since the template is rotated 360° with 1° steps in the horizontal direction on each and every pixel of the greyscale image. The only a priori knowledge used in this work is basics of the railroad corridor configuration including the gauge and the relative height of cables above the track bed. Such configurations are typically alike in the entire railroad corridor of a country and its neighboring countries so that the railroads of neighboring countries can be interconnected. Therefore, the developed algorithm can be applied to the railroad point clouds of an entire country as well as its neighboring countries that share similar railroad configurations.

Lastly, to investigate the local neighborhoods, a K-dimensional (KD) tree data structure [21] is usually constructed and a nearest neighbor search algorithm such as fast library for nearest neighbor (FLANN) [22] is employed. However, employing tree data structures for large datasets profoundly decreases the efficiency of the local neighborhood search. That is why a 2D grid is fitted to the data in the very first step of the methodology so that the computational efficiency of the neighborhood search is significantly enhanced.

## 6. Conclusions

This study proposes a novel method for automatic classification of urban railroad point clouds into three classes of rail track, contact cable, and catenary cable. The developed algorithm is able to classify point clouds with more complex configuration and lower point sampling while preserving a high computational efficiency. The methodology is enhanced by fitting a 2D grid to track bed for identification of rail track points and employing template matching to eliminate rail track false positives. Employing such hybrid data-driven and model-driven methodology takes advantage of data-driven approaches’ high computational efficiency and model-driven methods’ ability to handle poorly sampled datasets. This enables the algorithm to handle datasets with complex setup and low point sampling while keeping a high computational efficiency. This is tested on an urban railroad corridor point cloud whose sampling is lower than those utilized in Oude Elberink and Khoshelham [18] and whose configuration is more complex than in Arastounia [20]. The employed dataset covers 630 m of the Dutch urban railroad with four rail tracks, two contact cables, and two catenary cables. The dataset contains only geometrical information that is 3D coordinates of the points, with no intensity and no RGB data. The obtained results indicate that all objects of interest are separately recognized and the type of object is also identified. The contact cables achieve the best classification result due to their higher sampling rate and isolated position compared to other objects of interest in the railroad corridor. The catenary cables obtain the lowest precision due to the false positives at their intersections with masts. Moreover, the rail tracks’ high precision (98.4%) indicates the great impact of the employed model-driven (template matching) method in excluding the false positives while preserving a high computational efficiency. The results indicate that 100% precision and 100% accuracy at the object level and an average 97.3% precision and an average 97.7% accuracy at the point cloud level are obtained. In the developed algorithm, no assumption is made and no a priori knowledge is utilized regarding the location, orientation, or the curvature of the objects of interest.

## Figures and Tables

**Table 1:** Point Sampling on Various Parts of the Dataset.

Object	Sampling
Track bed (around inner rail tracks)	817 points per m^2^
Track bed (around outer rail tracks)	323 points per m^2^
Contact cable	17 points per m
Catenary cable	9 points per m

**Table 2:** The Classification Results at the Object Level.

Objects	Number of Objects	True Positives
Rail track	4	4
Contact cable	2	2
Catenary cable	2	2
All objects	8	8

**Table 3:** The Achieved Classification Results at the Point Cloud Level.

Objects	Precision (in %)	Accuracy (in %)
Rail track	98.4	96.5
Contact cable	99.2	98.8
Catenary cable	94.3	97.9
Average	97.3	97.7
